# A Two-Step Microwave Annealing Process for PAN Pre-Oxidation through a TM-Mode Cavity

**DOI:** 10.3390/polym13091476

**Published:** 2021-05-03

**Authors:** Yan-Ren Chen, Hsien-Wen Chao, Hung-Chun Hsu, Cheng-Hsuan Chan, Wei-Hsiang Lin, Che-Wei Tsai, Tsun-Hsu Chang

**Affiliations:** 1 Department of Materials Science and Engineering, National Tsing Hua University, 101, Section 2, Kuang Fu Road, Hsinchu 300044, Taiwan; himewako@banlii.tech (Y.-R.C.); ohyeahtw@yahoo.com.tw (W.-H.L.); 2 Department of Physics, National Tsing Hua University, 101, Section 2, Kuang Fu Road, Hsinchu 300044, Taiwan; s9822817@m98.nthu.edu.tw (H.-W.C.); z11452@yahoo.com.tw (H.-C.H.); rfmwfan@gmail.com (C.-H.C.)

**Keywords:** polyacrylonitrile, pre-oxidation, microwave annealing, carbon fiber, nonthermal effect

## Abstract

A novel microwave annealing system and a specific processing condition are proposed for the pre-oxidation of carbon fiber. The microwave annealing system consists of a TM-mode resonant cavity and a silicon carbide (SiC) susceptor. The TM-mode cavity enhances the electric field at the center. The SiC susceptor absorbs part of the microwave energy and converts it to heat. The enhanced fields and the SiC susceptor provide both nonthermal and thermal treatments for fibrous materials with various dielectric properties. Furthermore, a two-step microwave annealing process is used to oxidize polyacrylonitrile (PAN) fiber. The scanning electron microscopy (SEM) images, differential scanning calorimetry (DSC), and X-ray diffraction (XRD) results support the theory that the microwave annealing can achieve a high aromatic index of 66.39% in just 13 min, 9 times faster than the traditional processing time. The results of the Raman spectra also illustrate that the sheath-core factor of the microwave-heated specimen is closer to one than that of the conventional furnace-heated type, which agree with the images of the cross-section area.

## 1. Introduction

Carbon fiber reinforced polymers are advanced composite materials used in various applications, such as aerospace, automotive, and sports equipment [1,2,3]. Carbon fiber provides extreme strength when it is bound with plastic polymer resin in any shape. Besides, the properties of light weight and flexibility are both tremendous advantages of this composite material. Polyacrylonitrile (PAN) is the raw material of high-quality carbon fiber in a great deal of manufacturing nowadays. The PAN fiber goes through a series of chemical reactions in various processing stages and finally turns into the so-called carbon fiber. Pre-oxidation is the first stage of the entire process and also the most time-consuming part. Traditionally, the pre-oxidation process must be controlled under a slow temperature rise from 200 °C to 300 °C in about 2 h, while the carbonization process takes only 5−10 min. Nanoparticle coating and chemical modification on PAN fiber shortened the processing time [4,5,6,7]. Meng-Meng Qiao et al. have proposed that PAN pre-oxidation can be improved at 240 °C for 20 min through a pretreatment of immersion in a graphene oxide/PVA solution for 5 h and coating graphene oxide particles on the surface of the PAN fibers. On the other hand, Qin Ouyang et al. demonstrated a structural evolution on PAN by adding 1.5 mol% itaconic acid and promoting the stabilization reaction at 240 °C for 30 min by initiating ionic cyclization of nitrile groups. The pretreatment may take much more time for immersion or removing the aqueous solution.

Microwave annealing is a potential method to shorten the lengthy processing time. Microwave annealing features a lower processing temperature and a shorter processing time than the conventional heating methods for crystallization, chemical reaction, and phase transformation [8,9,10,11,12,13]. The annealed material is volumetrically penetrated and selectively heated by microwave. Hence, microwave annealing could eliminate the formation of a sheath-core structure for pre-oxidation of PAN, which is generated by the temperature gradient. Jian-Hua Liu et al. presented a method for oxidative stabilization of PAN by irradiating a 960 W microwave source of 2.45 GHz [14]. Although it shortened the processing time to 25 min and produced uniformity of element distribution in the radial direction, the unignorable energy cost is still an issue for practical applications. As an improvement on the design of the microwave heating system, a well-controlled electric field is required to enhance the nonthermal effect during microwave annealing.

This article reveals a microwave resonant cavity with a TM-mode of 2.45 GHz for PAN fiber’s pre-oxidation process. An improved E-field is employed in the cavity for enhancing the interaction between microwave and the annealed material with a lower energy cost [15,16,17,18]. The PAN fiber is then annealed under several processing conditions and analyzed by scanning electron microscopy (SEM) [19,20,21] and X-ray diffraction (XRD) [22,23,24,25]. An optimized process for microwave annealing would be finally determined by estimating the aromatic index values for every condition. The results show that raw PAN fiber can be pre-oxidized in 13 min by a two-step microwave annealing process.

## 2. Characteristics of a Well-Defined TM-Mode Resonant Cavity

**Figure 1:**
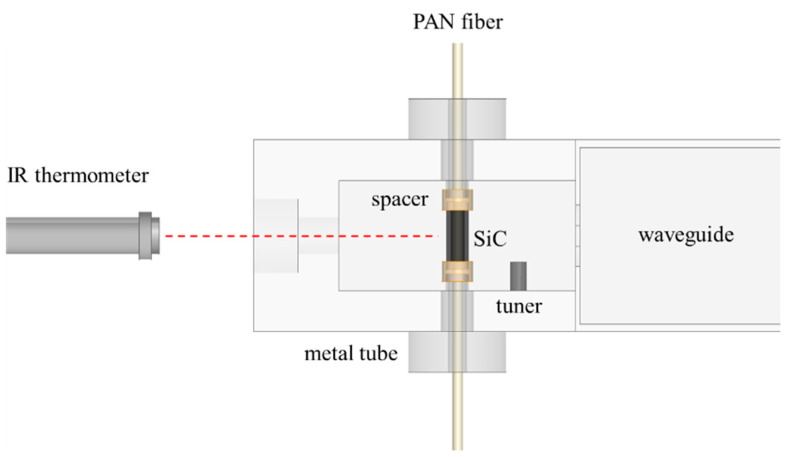
A side-view of the TM-mode resonant cavity for the pre-oxidation process of PAN fiber.

**Figure 2:**
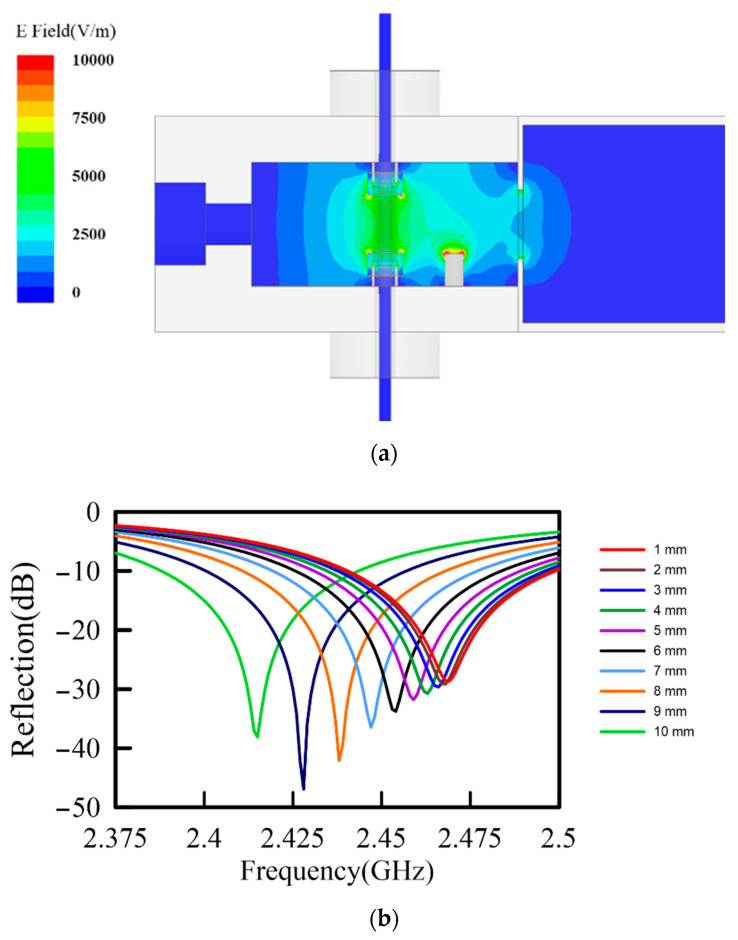
The simulation results of the TM-mode resonant cavity. (**a**) The distribution of E-field at microwave power of 20 W. (**b**) The frequency response of the cavity varied with the height of the tuner from 1 mm to 10 mm.

Figure 1 is the proposed microwave annealing system. This system consisted of an empty cavity, a microwave susceptor, two hollow ceramic spacers, and two hollow metal tubes. The cavity was made of metal with an inner dimension of 90 mm × 58 mm × 27 mm. Two hollow metal tubes were constructed at the middle-top and middle-bottom of the cavity to initiate a resonance of TM-mode. Figure 2a shows the electromagnetic simulation results, using a full-wave solver, high frequency structure simulator (HFSS, ANSYS Corp.). A well-controlled and enhanced E-field was defined between the two ends of the metal tubes since the strong boundary conditions were driven by the shape of the metal tubes.

A lane of PAN fiber with 12 K filaments went through the hollow metal tubes and susceptor for serial production. However, the color and dielectric properties of PAN fiber varied during the pre-oxidation process, which led to the change of the resonant frequency of the cavity. Furthermore, the frequency of the input microwave source (magnetron) was fixed at 2.45 GHz, the mismatch of input frequency and resonant frequency resulted in lower energy efficiency. An extra metal rod (tuner) was designed to solve the problem. Figure 2b shows the frequency response of the TM-mode resonant cavity. The peak position exhibited the resonant frequency of the cavity which was determined by the geometric size and the inside material properties. In our previous research, the dielectric constant of PAN varied from 2.82 to 6.50 after the pre-oxidation process, and the loss tangent went from 0.0074 to 0.8920, relatively. The variation of material properties resulted in a heavy shifting in the resonant frequency of the cavity. The tuner provided a controllable way to eliminate the mismatch between the input frequency from the magnetron and the resonant frequency of the cavity. The tuner also enhanced the microwave heating efficiency by lowering the power reflection.

**Figure 3:**
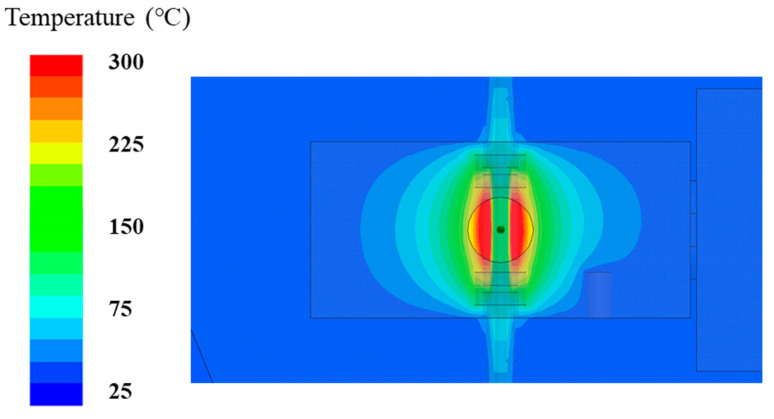
The simulation result of the TM-mode cavity’s thermal field with the microwave power = 20 W.

The microwave susceptor, made from silicon carbide (SiC), was a hollow tube of ϕ 3.5 mm × 16.5 mm connected to the metal tubes at the two ends through the ceramic spacers. The susceptor was employed for absorbing microwave energy and forming a uniform thermal field by its high dielectric loss and thermal conductivity. Figure 3 shows the simulation results of the thermal field. The thermal distribution existed in the region of the susceptor only. It was thermally insulated by the ceramic spacers at the two ends. Therefore, we defined the length of the susceptor as the processing region of the pre-oxidation and the time it took from one end to the other as the processing time. A window was located at the front side for the temperature measurement by an IR (infrared) thermometer. The IR thermometer pointed to the middle of the susceptor and measured its surface temperature as the processing temperature.

## 3. Specimen Preparation and Analysis

**Figure 4:**
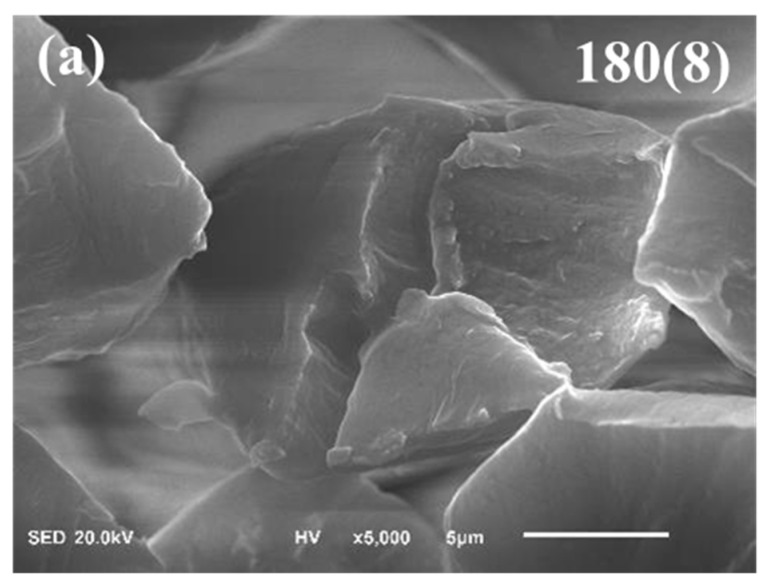

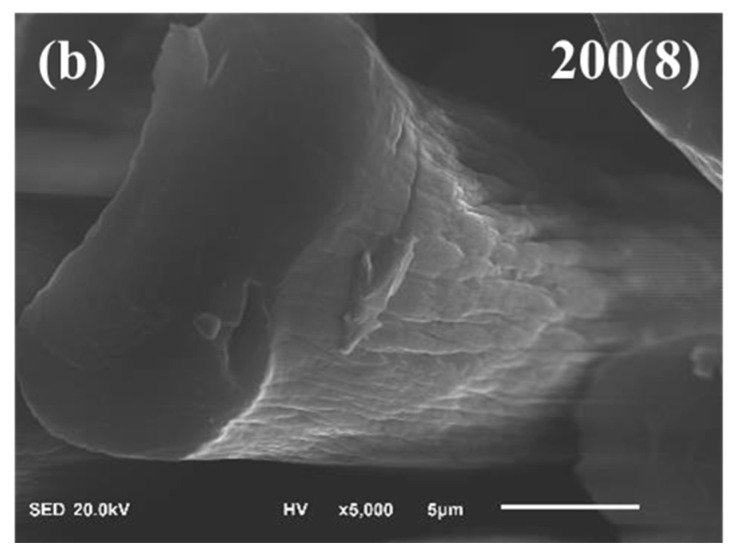
The cross-section view of the pre-oxidized specimen (**a**) 180(8) and (**b**) 200(8) using SEM.

Traditionally, the pre-oxidation of PAN fiber initiates at around 200 °C and is gradually accomplished with the rise in the processing temperature. The pre-oxidized specimens were measured by SEM and XRD. Then the values of the aromatic index were estimated to characterize the degree of the pre-oxidation. A lane of PAN fiber of 12 K filaments which was provided by Jinggong Technology went through the microwave annealing system under different processing conditions (temperature and time). The specimen’s name indicates the processing conditions. For example, 180(8) denotes that the PAN fiber moves 16.5 mm (the length of susceptor) with the surface temperature at 180 °C for 8 min. The lane of pre-oxidized PAN fiber looked dark and slightly burgundy through irradiation by an intense light source. Figure 4 shows the cross-section area of the pre-oxidized specimens from SEM, which was randomly picked from the lane. In Figure 4a, the cross-section area of specimen 180(8) features an uneven fracture surface, while specimen 200(8) in Figure 4b does not. The XRD results are introduced for a more detailed explanation.

**Figure 5:**
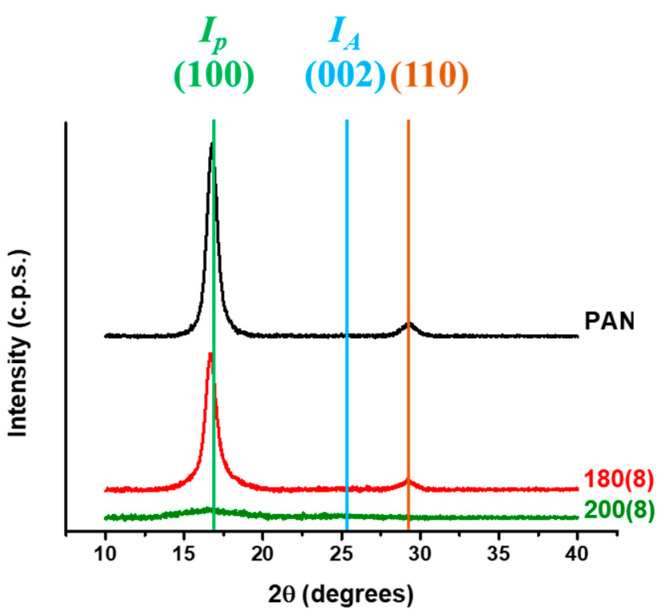
The variance of XRD results under different processing conditions.

Figure 5 reveals the variance of XRD results for the pre-oxidized specimens. The raw PAN fiber figured two characteristic peaks, one was (100) at 2θ = 17°, and the other was (110) at 2θ = 29°. These peaks implied the chemical structure of the fiber was a chain-shaped texture, and they would gradually diminish during the annealing process. Then they finally turned into a characteristic peak of (002) at 2θ = 25° that implied the formation of a ladder-shaped texture with the completion of the pre-oxidation. Specimen 180(8) indicated the chemical structure was still almost the same as the raw PAN fiber after the annealing process. Since PAN is a kind of thermoplastic chain-shaped polymer, the temperature effect makes it soften and fracture easily by shear force, and results in the uneven fracture surface appearing in Figure 4a. Furthermore, the (100) peak of specimen 200(8) diminished obviously, while the degree of the pre-oxidation was insufficient since the (002) peak was still not revealed.

Increasing the processing temperature would enhance the completion of the pre-oxidation. However, a significant variance in temperature makes the PAN fiber easy to break when the processing temperature is over 220 °C. To avoid severe deformation in the chemical structure in such a short time, therefore, we introduced a two-step microwave annealing process for PAN pre-oxidation.

## 4. Two-Step Microwave Pre-Oxidation Process of PAN Fiber

**Figure 6:**
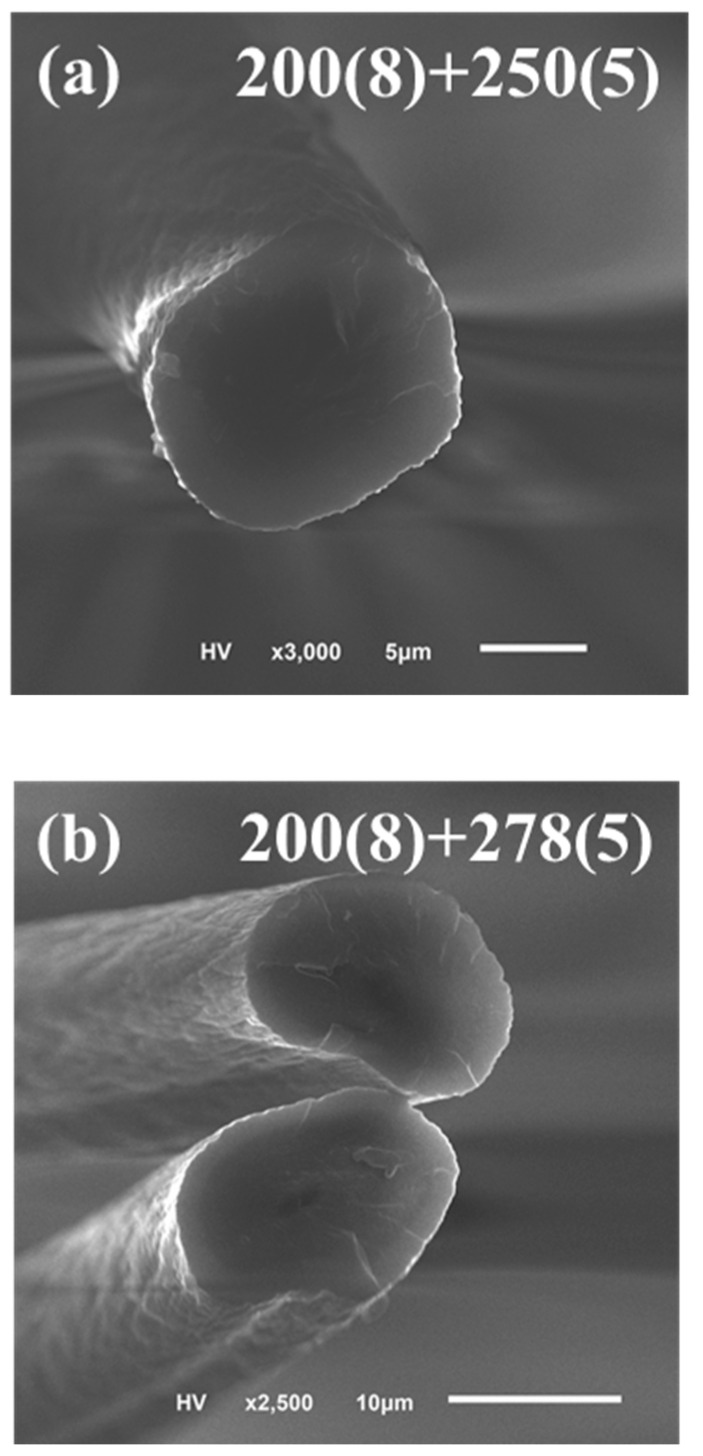

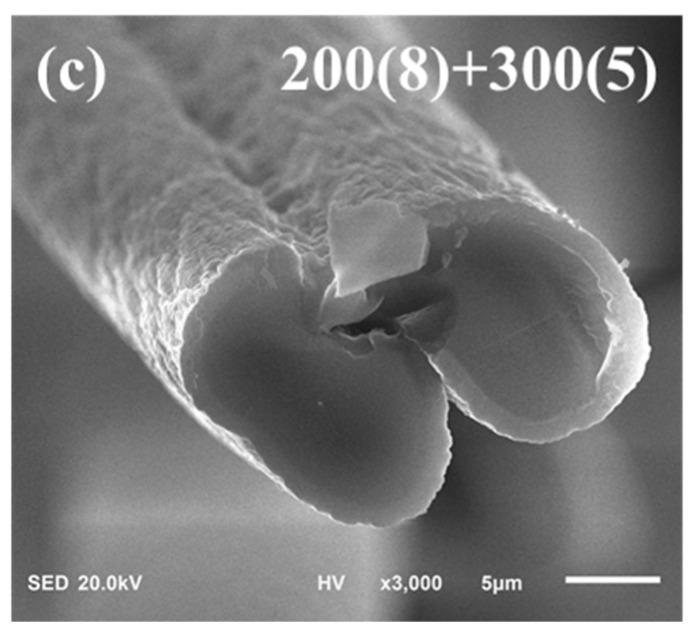
The cross-section view of the pre-oxidized specimens (**a**) 200(8) + 250(5) (**b**) 200(8) + 278(5) and (**c**) 200(8) + 300(5) using SEM.

This two-step microwave pre-oxidation processing had two purposes: (1) forming a metastable chemical structure for a minimum supporting strength at the first step with a lower temperature over a longer time, and (2) forming a very stable ladder-shaped structure at the second step with a higher temperature in a shorter time. Owing to the XRD results that feature in Figure 5, we defined 200 °C as the first processing temperature and combined with a higher temperature range from 250 to 300 °C as the second. The specimens with the two-step microwave annealing process looked as dark as the commercially available carbon fiber no matter whether the second-step processing temperature was 250 °C or 300 °C. It was difficult to tell these pre-oxidized specimens with the naked eye. Figure 6 exhibits the cross-section view of the two-step pre-oxidized specimens by SEM. In Figure 6a, the cross-section view of specimen 200(8) + 250(5) looked much smoother, and the microwave process left a uniform annealing effect without a sheath-core structure, while in Figure 6b,c, damage appeared at the edge of specimens 200(8) + 278(5) and became severe for the case of specimens 200(8) + 300(5). We found that the microwave-heated specimens were easily burned out at 325 °C and higher for the second-step pre-oxidation processing temperatures. Figure 6 shows that the actual surface morphology of the PAN fibers was also destroyed and stuck to each other even when the specimens were just heated at 278 °C and 300 °C. Therefore, we considered this damage as a kind of thermal fusion and that the polymer melted under the unexpected annealing temperature.

**Figure 7:**
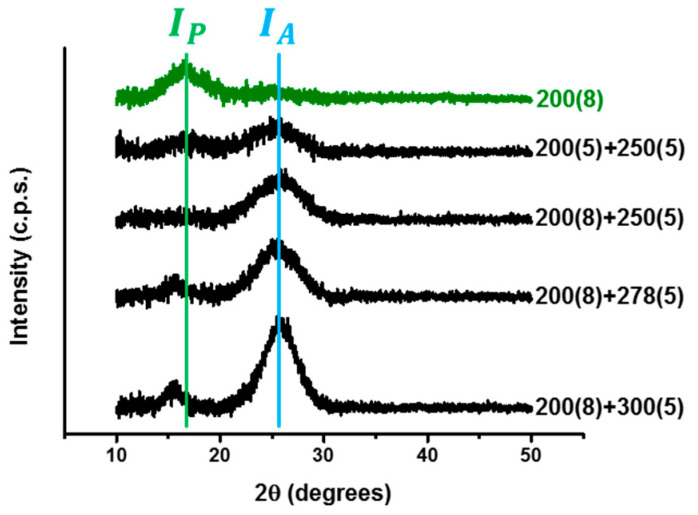
The XRD results of specimens under several two-step processing conditions.

Compared with Figure 5, the XRD results in Figure 7 demonstrate that the characteristic peak of (200) at 2θ = 25° was revealed after the two-step microwave annealing process. The higher the processing temperature at the seconds step, the better the crystallization of (200). Besides, the processing time at the first step would slightly affect the result. Using a shorter processing time, i.e., 5 min. would produce worse crystallization. The aromatic index of each specimen can be estimated from the XRD results and expressed as:

(1) $$\mathrm{Aromatic}\;\mathrm{Index}\;(\%)=\frac{I_{A}}{I_{A}+I_{P}}\times \;100\%$$

where *I_A_* is the area integration of (100) peak at 2θ = 17°, and *I_P_* is the area integration of (200) peak at 2θ = 25°. Table 1 shows the estimated values of the aromatic index by the XRD results. In the applications of industry, PAN fiber would be pre-oxidized to an aromatic index of 60–70% for use in the later process. Although the aromatic index values for the three processing conditions are applicable, the thermal fusion on the surface would result in expectable defects in the following process. To achieve a final product with fewer defects and better mechanical properties, we proposed that 200(8) + 250(5) would be the most suitable, two-step microwave processing conditions for the pre-oxidation of PAN fiber.

**Figure 8:**
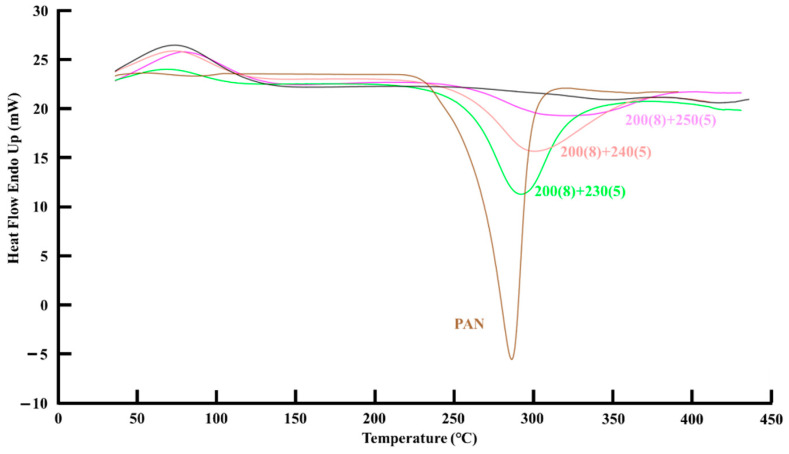
The DSC results for the specimens of the two-step microwave annealing process.

The DSC spectrum exhibited a coherent result as XRD. Figure 8 shows the DSC results for the two-step microwave pre-oxidation process. The area integration of exothermic peak (exothermic heat, ΔH) for each processing condition can be compared to the exothermic heat of raw PAN fiber and estimate the degree of aromatic reaction. It explores a trend that the exothermic peak gradually becomes weaker and broader when raising the processing temperature of the second-step annealing. The aromatic index from the DSC results can be estimated as follows:

(2) $$\mathrm{Aromatic}\;\mathrm{Index}\;(\%)=\left(1-\frac{\Delta H\;\mathrm{of}\;\mathrm{pre}-\mathrm{oxidized}\;\mathrm{fiber}\;}{\Delta H\;\mathrm{of}\;\mathrm{raw}\;\mathrm{PAN}\;\mathrm{fiber}}\right)\times \;100\%$$

Table 2 shows the estimations of the aromatic index related to the raw PAN fiber. The values of the aromatic index from DSC all were larger than from XRD, and it presented a finer processing condition for the second-step annealing process at 240 °C for 5 min.

## 5. Comparison with Conventional Heating Process

**Figure 9:**
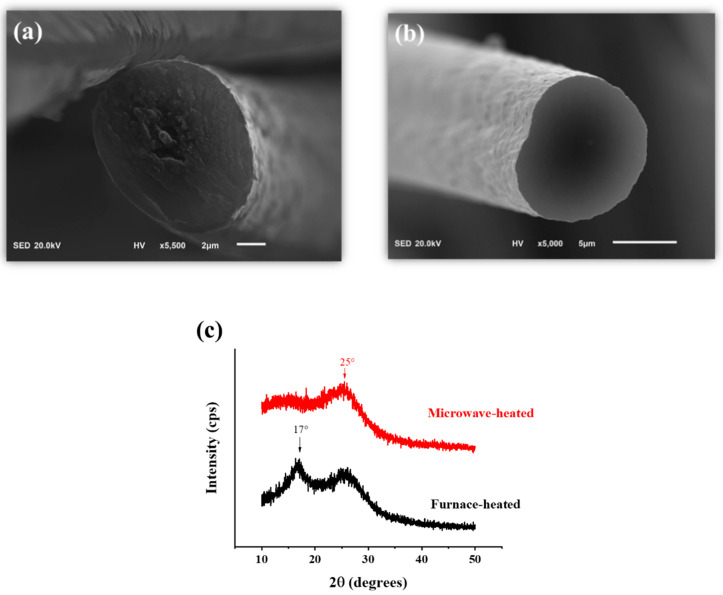
Comparison of furnace-heated and microwave-heated pre-oxidation specimens. (**a**) The cross-section area of the furnace-heated specimen. (**b**) The cross-section area of the microwave-heated specimen. (**c**) The XRD result reveals that the furnace-heated specimen still has a residual part of linear polymer structure.

Conventionally, the pre-oxidation process is controlled at a temperature range of 200−300 °C in the air with a slow rate of temperature rise for about 2 h. The conventionally pre-oxidized specimen was provided by TAIRYFIL (Tairylan, Formosa Plastics Corporation) which was heated in a box furnace with several temperature intervals and treated by the procedure mentioned above. Figure 9a is the SEM image from the cross-section area of the furnace-heated specimen. It featured a sheath-core structure and formed an uneven fracture at the cross-section area. Compared to Figure 9a,b shows the microwave-heated specimens 200(8) + 240(5). Obviously, the cross-section area did not show a sheath-core structure and it became much smooth than the furnace-heated one. The sheath-core structure results from the existing temperature gradient since the heat energy flux in a traditional furnace is from outer to inner, especially as most of them are electrothermic systems. Microwave heating is based on the interaction between microwave energy and material dielectric properties by volumetric penetration, the specimen is simultaneously heated in the radial direction, and the existing temperature gradient is much less than the traditional furnace. The XRD results in Figure 9c show that a part of the structure in the furnace-heated specimen was still a linear chain-like structure since the peak of (100) at 2θ = 17° remained, while the peak of (200) at 2θ = 25° determined that most parts of the structure in the microwave-heated specimen transformed into a ladder-like structure.

**Figure 10:**
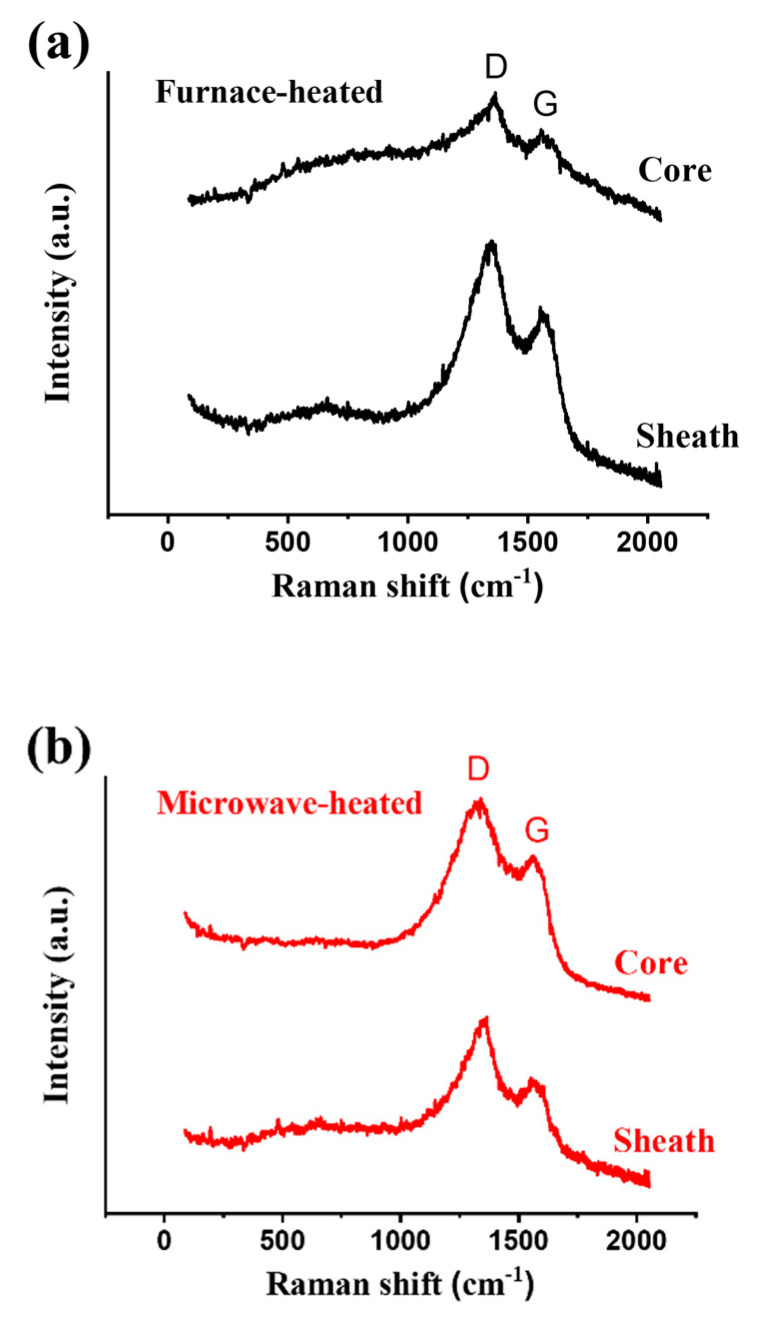
The Raman spectra of (**a**) furnace-heated and (**b**) microwave-heated specimens.

The Raman spectrum can help us to realize the numerical difference between the two of them. Figure 10 demonstrates the D-peak and G-peak at the sheath part and the core part for the two kinds of pre-oxidized fiber, and Table 3 shows the R values and the sheath-core factors. The R-value is the ratio of peak D and peak G, and the sheath-core factor is the ratio of the R-value of sheath and R-value of core, which expresses the degree of difference between the two parts:

(3) $$R=\frac{\mathrm{intensity}\;\mathrm{of}\;\mathrm{peak}\;D}{\mathrm{intensity}\;\mathrm{of}\;\mathrm{peak}\;G}$$

(4) $$S-C\;\mathrm{factor}=\frac{R\;\mathrm{of}\;\mathrm{sheath}}{R\;\mathrm{of}\;\mathrm{core}}$$

It provides convincing evidence that the microwave can achieve a more uniform processing effect since the sheath-core factor of the microwave-heated specimen was closer to 1.

## 6. Conclusions

In summary, we proposed a TM-mode resonant cavity as a microwave annealing system with an enhanced E-field for heating. Although the raw PAN is a low-loss dielectric material, the lengthy processing time of the pre-oxidation was shortened by a two-step microwave annealing process as the microwave power was only 20 W. The SEM images and XRD results have determined that PAN fiber could be pre-oxidized to an aromatic index of 66.39% after combining processing conditions of 200 °C for 8 min and 250 °C for 5 min. The DSC results also provided a finer processing condition for the second-step annealing.

Furthermore, a conventionally furnace-heated pre-oxidation specimen was compared to the microwave-heated one. The SEM images and XRD results presented an obvious difference in the cross-section area of the two specimens. The microwave-heated specimen presented a smooth fracture surface without sheath-core structure and a higher degree of crystallization on the (002) surface. Raman spectra gave a numerical index for determining the uniformity of material properties after the pre-oxidation process of the two. It has proved that the non-thermal effect of microwave annealing can achieve a more uniform processing effect in a shorter processing time for pre-oxidation and might have great potential for industrial applications to lower the production cost of carbon fiber.

## Figures and Tables

**Table 1:** The aromatic index of specimens under several processing conditions from XRD results.

Specimens	Aromatic Index (%)
PAN	-
180(8)	N/A
180(8) + 250(5)	51.07
180(8) + 300(5)	65.45
200(8)	25.31
200(5) + 250(5)	57.48
200(8) + 250(5)	66.39
200(8) + 278(5)	70.90
200(8) + 300(5)	71.36

**Table 2:** The aromatic index of specimens under several processing conditions from DSC results.

Specimens	ΔH(J)	AI(%)
PAN	803	-
200(8) + 230(5)	440	45.3
200(8) + 240(5)	319	60.3
200(8) + 250(5)	136	83.1

**Table 3:** The sheath-core factor of furnace-heated and microwave-heated specimens.

Specimens	R of Sheath	R of Core	S-C Factor
furnace-heated	1.3	1.15	1.13
microwave-heated	1.24	1.33	0.93

## Data Availability

The data presented in this study are available on request from the corresponding author.
